# Genetic and Phenotypic Profiling of Triptan Users in a Swedish Cluster Headache Cohort

**DOI:** 10.1007/s12031-024-02219-1

**Published:** 2024-04-18

**Authors:** Felicia Jennysdotter Olofsgård, Caroline Ran, Yuyan Qin, Carmen Fourier, Elisabet Waldenlind, Anna Steinberg, Christina Sjöstrand, Andrea Carmine Belin

**Affiliations:** 1 Centre for Cluster Headache, Department of Neuroscience, Karolinska Institutet, Stockholm, Sweden; 2 Department of Clinical Neuroscience, Karolinska Institutet, Stockholm, Sweden; 3 Department of Neurology, Karolinska University Hospital, Stockholm, Sweden; 4 Department of Neurology, Danderyd Hospital, Stockholm, Sweden

**Keywords:** rs5443, rs1024905, rs6724624, rs2651899, 5-HTTLPR

## Abstract

Up to 25% of individuals who live with cluster headache (CH), an extremely painful primary headache disorder, do not adequately respond to the first-line treatment, triptans. Studies have indicated that genetic variants can play a role in treatment response. Likewise, differences in clinical characteristics can give clues to mechanisms underlying triptan non-response. Our aim was to investigate five genetic variants previously implicated in triptan response and their relation to triptan usage in our Swedish CH cohort and to investigate potential distinctions in clinical characteristics. 545 CH patients were screened for the genetic variants rs1024905, rs6724624, rs4795541, rs5443, and rs2651899 with a case control design based on triptan usage. Analysis of clinical characteristics was based on self-reported questionnaire data from 893 patients. One genetic variant, rs1024905, was significantly associated with triptan non-usage in CH (*Pc* = 0.010). In addition, multi-allele effector analysis showed that individuals with a higher number of effector variants were less likely to use triptans (*P* = 0.007). Analysis of clinical characteristics showed that triptan users were more likely to have alcohol as a trigger (57.4% vs 43.4%, *P* = 0.002), have autonomic symptoms (95.1% vs 88.1%, *P* = 0.002), and be current smokers (27.0% vs 21.9%, *P* = 0.033) compared to non-users. These results support the hypothesis that genetic variants can play a role in triptan usage in CH and that patients with a typical CH phenotype are more likely to use triptans.

**Supplementary Information:**

The online version contains supplementary material available at 10.1007/s12031-024-02219-1.

## Introduction

Cluster headache (CH) is considered the most painful disorder known to man, with a prevalence of 0.1% in the general population (May et al. 2018). Though not curative, there is medication available for CH both in the form of abortive medication and prophylactic treatment. Triptans are a first-line treatment for CH attacks and are seen as the most effective abortive medication. Triptans bind as agonists with high affinity to serotonin/5-hydroxytryptamine (5-HT)_1B/1D_ receptors. Upon activation these receptors cause vasoconstriction in intracranial blood vessels and inhibition of neuropeptide release in the trigeminal nerve (Nicolas and Nicolas 2022). This includes the inhibition of calcitonin gene-related peptide (CGRP) release in trigeminal nerve endings as proven by animal models (Arvieu et al. 1996). In human subjects, subcutaneous injections of triptans taken at the start of a CH attack are reported to achieve pain relief within 15 min in 75% of the CH study participants (Ekbom et al. 1993). Intranasal administration of triptans can also be effective, but to a lesser extent with only 57% of the patients reporting pain relief 30 min after administration (van Vliet et al. 2003). These data indicate that a considerable percentage of CH patients do not respond to triptans as aspired. The mechanism behind the unresponsiveness and/or delayed response is still unclear.

Previously, multiple studies have found links between genetic variants and triptan response in both CH and migraine patients. However, many of the reported associations were weak and not thoroughly established. Schürks et al. investigated the association between triptan response in CH and the single nucleotide polymorphism (SNP) rs5443 situated in the guanine nucleotide-binding protein subunit beta-3 (*GNB3*) gene. The *GNB3* gene translates to a subunit of the intracellular protein, heterotrimeric guanine nucleotide-binding protein (G-protein), which forms a complex with G-protein coupled receptors and helps with signal transmission. Schürks et al. found the heterozygote genotype (C/T) to be more common in triptan responders as opposed to the homozygous wildtype carriers (C/C) (Schürks et al. 2007a). Papasavva et al. found a similar trend in a Greek CH cohort; however, the association did not reach significance (Papasavva et al. 2020). The rs5443 variant results in a change in the splicing of the *GNB3* gene which leads to a shorter protein variant, Gβ3s, which is associated with increased receptor signaling (Genecards 2022a). Since triptans bind to G-protein coupled receptors, the increased receptor signaling seen with Gβ3s could hypothetically increase the effect of the drug.

The well-studied 5-HT transporter gene-linked polymorphic region (*5-HTTLPR*) indel variant, represented by rs4795541, sits in the promoter region of the serotonin transporter gene. It has been linked to several conditions including mood disorders, stress response, response to selective serotonin reuptake inhibitors (SSRIs), and the S allele has been linked to non-response in CH (Lesch et al. 1996; Smeraldi et al. 1998; Markus and Firk 2009; Schürks et al. 2014; Ren et al. 2020; Jang et al. 2021). The more common “long” allele (L) of this variant consists of a 43 bp indel which the “short” allele (S) lacks. The L allele promotes increased transcription of the serotonin transporter gene as compared to the S allele. Furthermore, the L allele lies in close proximity to a SNP, rs25531 (A > G (L_A_, L_G_), whose G allele (L_G_) is shown to decrease the transcriptional levels to nearly that of the S allele (Hu et al. 2006).

An Italian study on migraine found additive effects of the intergenic variants rs1024905 and rs6724624, located on chromosome 12 respectively chromosome 2, to be associated with triptan response in patients with migraine (Cargnin et al. 2019). A genome-wide association study (GWAS) migraine SNP, rs2651899, located in the PR/SET Domain 16 (PRDM16) gene, was associated with triptan response in a Danish migraine cohort (Christensen et al. 2016). However, a later study could not find similar associations for any of these variants (Petersen et al. 2023).

Differences in clinical features can likewise give clues as to biological mechanisms behind non-response to treatment and CH pathology. A few reports have highlighted the disease characteristics in triptan responders and non-responders in CH. Giani et al. studied the differences in disease characteristics between these two groups (Giani et al. 2021). They found non-responders to have a higher attack frequency and longer attack duration. Petersen et al. found that episodic CH patients were more likely to respond to triptans than chronic CH patients (Petersen et al. 2023).

The overall aim of this project was to analyze genetic variants with proposed associations to triptan response, in a large CH patient cohort, and to explore potential disease characteristics behind non-response in a case-control manner. This will hopefully lead to a better understanding of the underlying factors contributing to triptan non-response and ultimately help identify patients at greater risk for ineffective triptan treatment. To achieve this, we screened and investigated the association between five genetic variants and usage of triptans in our Swedish CH cohort and compared relevant clinical characteristics. Regular triptan use was applied as a parameter to model triptan response in our study as triptan response data was not available.

## Methodology

### Patient Information

The material consisted of 893 study participants (Table 1) diagnosed with CH and subtype by a neurologist according to the criteria of International Classification of Headache Disorders (ICHD), 3^rd^ edition (Olesen 2018). Study participants were recruited from throughout Sweden in collaboration with the neurology clinic at Karolinska University Hospital from 2014 to 2022. The study was approved by the Swedish Ethical Review Authority in Stockholm (diary number 2014/656-31/4). Written informed consent was obtained from all study participants. All experiments were conducted in accordance with the declaration of Helsinki adopted by the World Medical Association in regard to human samples. Upon recruitment to our biobank (described in Steinberg et al. 2018), participants were asked to give a blood sample and fill out a questionnaire involving questions regarding disease characteristics, lifestyle, and family history (Fourier et al. 2023). DNA was extracted from whole blood samples using standard protocols.

**Table 1:** Demographics

	**All participants with CH**	**Triptan users (injections or nasal spray)**	**Triptan non-users**	**Triptan tablet users (exclusively)**
**Number of individuals**	893	692	160	41
**Age (years)**	50.3 ± 14.3	49.4 ± 13.9	53.5 ± 15.2	51.9 ± 16.6
**Age at onset (years)**^**a**^	31.8 ± 13.4	31.0 ± 13.0	35.1 ± 14.7	34.4 ± 14.8
**Male % (*n*)**	65.6% (586)	67.8% (469)	58.8% (94)	56.1% (23)
**Chronic % (*n*)**^**b**^	12.5% (111)	12.3% (85)	12.1% (19)	17.1% (7)
**Heredity % (*n*)**^**c**^	12.4% (107)	13.1% (88)	8.3% (13)	15.4% (6)

Numerical data presented as mean ± standard deviation. Triptan users regularly use one or more of the following: sumatriptan injections, sumatriptan nasal spray, or zolmitriptan nasal spray. Triptan tablet users exclusively use triptans in the form of tablets: rizatriptan tablet, sumatriptan tablet, zolmitriptan tablet, and eletriptan tablet. Triptan non-users do not use triptans in any form. Percentages do not include missing data

*CH* cluster headache

^a^Age at onset (*n* =803)

^b^Info on chronic or episodic subtype from *n* = 888

^c^Info on heredity from *n* = 865

Study participants were grouped depending on their self-reported triptan usage. Triptan usage was classified as individuals taking one or multiple of the following: sumatriptan injections, sumatriptan nasal spray, or zolmitriptan nasal spray. The triptan tablet group included individuals who took triptans only in the form of tablets: rizatriptan, sumatriptan, zolmitriptan, and/or eletriptan tablet. Triptan tablets are rarely used in treatment of CH due to their slow-acting pharmacological effect (Brandt et al. 2020). To ensure groups were clearly defined, we excluded tablet-only users from the analysis. Triptan non-users were defined as the remaining individuals who did not take triptans in any form but had answered the survey. Nine of the triptan non-users took ergotamine.

### qPCR of rs5443, rs1024905, rs6724624, and rs2651899

TaqMan^®^ Quantitative Real-Time PCR (qPCR) was used to determine the allele frequency of rs5443, rs1024905, rs6724624, and rs2651899 with TaqMan genotyping assays (Online Resource 1, Table S1) and TaqMan Genotyping MasterMix (Thermo Fischer Scientific, Waltham, USA). qPCRs were conducted using a 7500 Fast Real-Time PCR system (Applied Biosystems, Foster City, CA, USA) according to the recommended protocol with slight modifications; 0.5X of SNP assay, 42 PCR cycles for rs1024905 and rs5443. The 7500 software version 2.0.6 was used for allelic discrimination. Genotype data for rs2651899 from 61.4% of the samples were obtained from a previous publication (Ran et al. 2018).

### PCR of 5-HTTLPR indel

A polymerase chain reaction (PCR) and restriction fragment length polymorphism method (PCR-RFLP) with *BcnI* cutting as developed by Schürks et al. (Schürks et al. 2014) was used to genotype the *5-HTTLPR* indel (rs4795541) and the accompanying SNP, rs25531. PCR was performed using previously published primers for the *5-HTTLPR* variant (Ellerbrock et al. 2021), obtained from Thermo Fisher Scientific. The Mastermix was composed of 0.2 μM forward and reverse primers (Thermo), 1x PCR buffer with (NH_4_)_2_SO_4_ (Thermo), 0.2 mM dNTP (Sigma, Saint Louis, USA), 1 mM Mg^2+^ (Thermo), and 0.5 U Taq DNA Polymerase recombinant (Thermo) in RNAse free H_2_O. Each reaction contained 1 μl DNA and 24 μl Mastermix.

The PCR reaction was conducted on a PTC-200 Peltier Thermal Cycler, (Conquer Scientific, San Diego, California) with the following cycling conditions also retrieved from Ellerbrock et al. (Ellerbrock et al. 2021) with slight modifications; 95°C for 10 min, 95°C for 30 s, 60°C for 30 s, and 72°C for 5 min, repeat 35x, elongation step at 72°C for 5 min. The PCR products were run on a 3.5% agarose gel (3.5% agarose (Thermo), 0.008% GelRed DNA Stain (Biotium, Fremont, USA)), at 70 V for 150 min using BioRad PowerPac (Thermo). For each sample, 10 μl of the PCR product was combined with 17 μl RNAse free H_2_O, 2 μl 10x FastDigest Buffer (Thermo) and 1 μl *BcnI* enzyme (Thermo), incubated at 37°C for 60 min then at 80°C for 20 min, and ran on a 3% agarose gel at 70 V for 120 min to determine the rs25531 genotype (L_A_ = 126 bp, 62 bp, 341 bp; L_G_ = 126 bp, 62 bp, 174 bp, 167 bp; S = 126 bp, 62 bp, 298 bp).

### Sequencing

Eighteen samples classified as having a L_G_S genotype (126 bp, 62 bp, 174 bp, 167 bp, and 298 bp) exhibited an extra band (341bp) when running the PCR gel and were therefore sent for Sanger sequencing to verify the genotype at the KIGene facility (Stockholm, Sweden). An additional nine samples with different genotypes were sequenced as positive controls and to verify the correctness of the results.

### Clinical Features Analysis

Clinical features data were obtained from the surveys filled out by patients when recruited to our biobank. For questions regarding attack frequency, attack duration, period duration, and period frequency, some participants filled in multiple answers. For those instances we kept the answer that was most severe (highest attack frequency, longest period duration, etc.). Missing data was not included in the final percentages or analysis. Response rate for clinical data can be found in Tables 1 and 3. Some quantitative variables were grouped evenly in the questionnaire to facilitate better readability for the participants. Heredity was defined as patients having one or more first, second, or third degree relative with CH.

### Statistical Analysis

**Fig 1:**
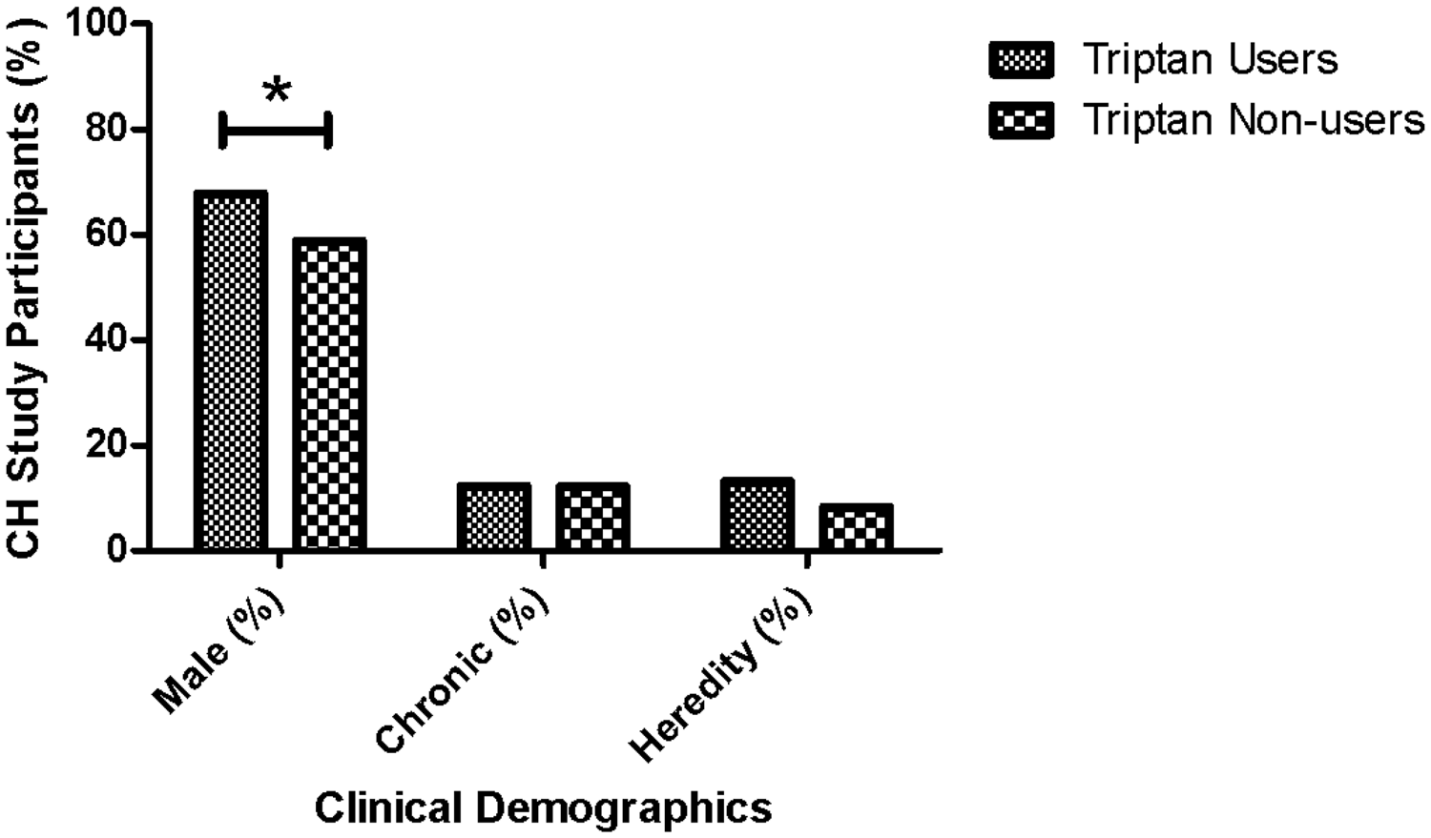
Sex, chronicity, and heredity based on triptan usage in CH patients. The figure shows an increased usage of triptans in males as compared to female CH patients. There is no difference in CH type (episodic vs chronic) and no difference in percentage of individuals with an affected relative (heredity) between triptan users and triptan non-users

Statistical analysis was conducted using Rstudio 4.1.1 (RStudio Team 2020) and PLINK 1.90 (Chang et al. 2015). Figure 1 was created in GraphPad Prism 5. Categorical data was presented as percentages and numerical data as mean ± standard deviation. Chi-square analyses and Wilcoxon test were used for statistical analysis of phenotypic data. Genetic association was analyzed using logistic regression under an additive model with sex as a covariate. The control group was defined as individuals taking triptans while the triptan non-users were classified as the case group for the logistic regression analysis since our main interest were factors that could lead to patients not using triptans. A two-tailed *P*-value of 0.05 was deemed significant. Bonferroni correction was applied for genetic testing.

A genetic effector score analysis was conducted using a non-weighted additive model. Effector alleles related to triptan non-usage/non-response were identified in our study, as well as in the literature (Online Resource 1, Table S1) (Schürks et al. 2007a, 2014; Christensen et al. 2016; Cargnin et al. 2019; Papasavva et al. 2020). The effector allele was defined as the allele more common in triptan non-users compared to users. For *5-HTTLPR,* the S allele was identified as the effector allele while both L_G_ and L_A_ were considered to be the non-effector alleles in a bi-allelic manner. The reported effector allele was equivalent for all SNPs except rs6724624 (Online Resource 1, Table S1); the major allele of rs6724624, C, was more common in non-users in our study, while the minor allele, G, was more common in triptan non-responders with migraine (Cargnin et al. 2019). Since our study included a substantially larger cohort than the Italian migraine study and considering our focus is CH, we conducted the genetic effector score analysis using C as the effector allele for rs6724624. A cumulative effector score for the five variants was attributed to each individual depending on the number of alleles they carried and compared using logistic regression with the effector score as a continuous variable and sex and age as covariates to account for bias. Individuals with missing genotypes for any of the variants were excluded from the analysis (remaining *n* = 489).

PS Power and Sample Size Calculation program Version 3.0 (Dupont and Plummer 1990) was used for power analysis. With a sample size of 518 CH patients, and the minor allele frequencies (MAFs) of rs1024905, rs6724624, and rs2651899, reported for Europeans in the 1000 Genomes Project Phase 3 and gnomAD exomes database (rs5443) (Ensembl genome browser 2022b), we have 80% power to detect an association with 0.522 < odds ratio (OR) > 1.885 for rs1024905, with 0.340 < OR > 2.082 for rs6724624, with 0.513 < OR > 1.876 for rs2651899 and with 0.466 < OR > 1.901 for rs5443. For the power calculation of 5-HTTLPR we used the MAF (S allele) from an article genotyping a European American population (Odgerel et al. 2013) which gave 80% power to detect 0.513 < OR > 1.876.

## Results

### Genetic Association Analysis

A total of 545 study participants diagnosed with CH were genotyped with qPCR with a call rate >98%. The call rate for *5-HTTLPR* genotyped with PCR-RFLP was 97.6%. All SNPs and the *5-HTTLPR* variant were in Hardy-Weinberg equilibrium (HWE).

Of the genotyped CH patients 75% (409 study participants) were categorized as triptan users and 20% (*n* = 109) as non-users, the rest of the study participants took triptans only in the form of tablets and were excluded from the analysis. Statistical analysis showed that the minor allele of rs1024905 was significantly more prevalent among non-users (*P* = 0.002, *P*_*c*_ = 0.010) (Table 2). The three remaining SNPs, rs5443, r6724624, and rs2651899, were equally distributed in the two groups (Table 2).

**Table 2:** Allele distributions of rs1024905, rs6724624, rs5443, rs2651899, and *5-HTTLPR* comparing triptan users vs non-users in a Swedish CH cohort

**Genetic variant**	**Allele**	**Triptan users % (*n*)**	**Triptan non-users % (*n*)**	**OR (95% CI)**	***P*-value**	***P***_***c***_**-value**
rs1024905	G	53.4% (435)	41.6% (89)	1.609 (1.19–2.18)	0.002	0.010
	C	46.6% (379)	58.4% (125)			
rs6724624	C	78.9% (634)	83.6% (179)	0.733 (0.49–1.10)	0.129	0.645
	G	21.1% (170)	16.4% (35)			
rs5443	C	71.1% (577)	71.0% (152)	1.014 (0.73–1.42)	0.933	> 1.0
	T	28.9% (235)	29.0% (62)			
rs2651899	T	58.9% (475)	59.3% (128)	0.986 (0.73–1.34)	0.929	> 1.0
	C	41.1% (331)	40.7% (88)			
5-HTTLPR Bi-allelic	L	58.6% (465)	50.9% (110)	1.342 (1.00–1.80)	0.048	0.240
	S	41.4% (329)	49.1% (106)			
5-HTTLPR^a^ Tri-allelic	L_A_	52.3% (415)	46.8% (101)	1.240 (0.92–1.67)	0.158	0.790
	L_G_ and S	47.7% (379)	53.2% (115)			

*CH* cluster headache, *5-HTTLPR* serotonin transport promotor polymorphism,* L* long allele,* S* short allele, *OR* odds ratios, *CI* confidence interval,* P*_*c*_*-value* Bonferroni corrected *P*-value

The genotypic distribution of *5-HTTLPR* was analyzed both as a bi-allelic and tri-allelic variant. For the bi-allelic analysis, only the presence (L) or absence (S) of a 43 bp insertion was investigated. Analysis showed the S allele being more common in triptan non-users though it did not hold after correcting for multiple comparisons (OR = 1.342, *P* = 0.048, *P*_*c*_ = 0.240) (Table 2). The tri-allelic analysis of *5-HTTLPR* included the rs25531 SNP (L_G_) located in proximity to the L allele. In this analysis the L_G_ allele was classified to be the same as the S allele because of previous data showing similar levels of gene expression. With this model, the trend for association for the S allele was not replicated (OR = 1.24,* P* = 0.158) (Table 2).

### Genetic Effector Score Analysis

To determine if there was an additive effect of carrying multiple effector alleles for triptan non-usage, we performed a genetic effector score analysis. Analyzing the sum effector score as a continuous variable for each individual showed a significant association between effector score and triptan usage (*P* = 0.007) with an estimate coefficient of 0.204. Sex (*P* = 0.04) and age (*P* < 0.001) were also significantly linked to triptan usage with an estimate coefficient of −0.483 for males and 0.031 for age.

### Clinical Features

Clinical features were investigated to see if there was a difference in disease presentation between triptan users and non-users. Phenotype data was available for 692 triptan users and 160 non-users (Table 1). The male to female ratio was more elevated in triptan users than in non-users (males: 67.8% vs 58.8%, *P* = 0.037) (Fig. 1). Triptans users were significantly younger than triptan non-users (*P* < 0.001) and had a younger age at onset (*P* = 0.003) (Table 1). There was no difference in CH subtype distribution (*P* = 1) or heredity (*P* = 0.130) between the groups (Fig 1, Table 1). Triptan users were more likely to have autonomic symptoms accompanying headache attacks (*P* = 0.002). Attack frequency also significantly differed (*P* < 0.001) with triptan users trending towards having more attacks per day (Table 3). However, attack duration, period duration, severity score (Steinberg et al. 2018), and duration of disease were not significantly different between the groups (Table 3). The overall presence of headache triggers was similar between triptan users and non-users. Nevertheless, triptan users more often reported to have alcohol as a trigger (*P* = 0.002). A higher proportion of triptan users were current smokers, whereas triptan non-users were more commonly former smokers (*P* = 0.033) (Table 3).

**Table 3:** Clinical features analysis comparing triptan users vs non-users in a Swedish CH cohort

	**Triptan users** **% (*n*)**	**Triptan non-users** **% (*n*)**	***P*-value**
**Attack frequency (attacks per day)**^**a**^ ***n* = 835**	5.1% (35) <1 40.6% (279) 1–2 39.5% (272) 3–5 14.8% (102) >6	13.6% (20) <1 44.2% (65) 1–2 31.3% (46) 3–5 10.9% (16) >6	< 0.001
**Attack duration (minutes)**^**a**^ ***n* = 830**	14.0% (95) 15–30 min 53.0% (360) 30–120 min 18.1% (123) 120–180 min 14.9% (101) >180 min	17.2% (26) 15–30 min 47.7% (72) 30–120 min 17.2% (26) 120–180 min 17.9% (27) >180 min	0.505
**Period duration (months)**^**a**^ ***n* = 817**	27.0% (182) 0–1 m 30.5% (205) 1–2 m 21.0% (141) 2–4 m 7.4% (50) 4–7 m 5.2% (35) 7–12 m 8.9% (60) >12 m	34.0% (49) 0–1 m 27.1% (39) 1–2 m 20.1% (29) 2–4 m 3.5% (5) 4–7 m 6.3% (9) 7–12 m 9.0% (13) >12 m	0.354
**Severity score**^**a,c**^ ***n* = 766**	7.3% (46) 2–4S 46.3% (290) 5–6S 37.4% (234) 7–9S 8.9% (56) >10S	8.6% (12) 2–4S 42.9% (60) 5–6S 36.4% (51) 7–9S 12.1% (17) >10S	0.619
**Occurrence of autonomic symptoms**^**a**^ ***n* = 838**	95.1% (653) yes 4.9% (34) no	88.1% (133) yes 11.9% (18) no	0.002
**Smoking status**^**a**^ ***n* = 848**	27.0% (186) current 40.6% (279) previous 32.4% (223) never	21.9% (35) current 51.9% (83) previous 26.3% (42) never	0.033
**Alcohol as a trigger?**^**a**^ ***n* = 824**	57.4% (386) yes 42.6% (286) no	43.4% (66) yes 56.6% (86) no	0.002
**Duration of disease (years)**^**b**^ ***n* = 768**	18.3 ± 13.7 years	17.3 ± 14.5 years	0.238
**Specific triggers**^**a**^ ***n* = 823**	56.0% (376) yes 44.0% (296) no	47.7% (72) yes 52.3% (79) no	0.080

*m* months, *S* severity score

^a^Chi-square test used

^b^Wilcoxon test used

^c^Severity score as described in “Cluster headache – clinical pattern and a new severity scale in a Swedish cohort” (Steinberg et al. 2018)

## Discussion

As a first-line treatment, triptans offer relief from an excruciatingly painful disorder. Understanding the mechanisms behind triptan non-responsiveness and identifying CH patients most at risk for inconsistent response would be of benefit for CH treatment moving forward. We hypothesize that factors such as genetic variations may affect triptan efficiency. As suggested by Giani et al. the variation of triptan response between CH patients can also indicate differences in disease mechanisms and phenotype (Giani et al. 2021).

The SNP rs1024905 is significantly associated with triptan usage (*P*_*c*_ = 0.010) with an OR of 1.609 and the major allele of rs6724624 shows a weak trend towards triptan non-usage (*P* = 0.129, *P*_*c*_= 0.645) (Table 2). Cargnin et al. found rs1024905 in conjunction with rs6724624 to be linked with triptan response in migraine (Cargnin et al. 2019). The C allele of rs1024905, associated with reduced likelihood of using triptans, has been linked to lower RNA expression of several genes including Chromosome 12 Open Reading Frame 4 (*C12orf4*) in the tibial artery compared to the G allele (GTEx Portal 2022c). C12orf4 has a function in mast cell degranulation and, though evidence remains contradictory, the potential involvement of mast cells in CH has long been discussed (Mathew 1998; Mazuc et al. 2014; Dimitriadou et al. 2016; Pellesi et al. 2022). For rs6724624 the allele frequencies in our Swedish cohort are in opposition with the previously published report where the minor G allele was identified as a risk factor for inconsistent response (Cargnin et al. 2019). This could be due to a difference between the two diseases investigated and/or the methods used. Cargnin et al. focused on triptan response in migraine, while our study looked at triptan usage in CH. Finding a significant association between rs1024905 and triptan response/usage in both CH and migraine could indicate similar mechanisms for triptans in both diseases.

The *GNB3* variant rs5443 was not significantly associated with triptan usage. Schürks et al. found the C/T genotype to be more prevalent in triptan responders as opposed to the C/C genotype (Schürks et al. 2007a). We additionally performed a genotypic logistic regression analysis with sex as a covariate for rs5443 to compare results, but we could not confirm the association found by Schürks et al. (*P* = 0.297).

The short allele of the *5-HTTLPR* genetic variant showed a trend towards being more common in patients not using triptans (bi-allelic: *P* = 0.048, tri-allelic: *P* = 0.158) (Table 2). The deletion of the 43 bp region leads to a decrease in transcriptional levels of the serotonin transporter gene. This in turn lowers the amount of serotonin that gets pumped back in the presynaptic terminal and therefore increases the amount in the synaptic cleft. Schürks et al found a non-significant trend for the tri-allelic SS genotype in triptan non-responders which is in line with our results (Schürks et al. 2014). They argue that it could indicate that clearance of serotonin in the synaptic cleft could be important for the efficacy of triptans (Schürks et al. 2014). In contrast to Schürks et al. the bi-allelic variant in our study showed a greater association with triptan usage than the tri-allelic variant which includes the L_G_ and L_A_ alleles. Consistent with our data, a recent meta-analysis evaluating the link between the *5-HTTLPR* polymorphism and SSRI response in major depressive disorder, found that the bi-allelic L allele was associated with better response to SSRIs in Caucasian populations, but not the tri-allelic variant, which may suggest that the effect of the L_G_ variant was previously overestimated (Ren et al. 2020). It can be worth noting that the patient population of this study is thrice larger than the study previously performed on CH.

The cumulative effect of multiple polymorphisms may reveal more substantial differences than analyzing individual variants. Therefore, a genetic effector score for each individual was calculated. This showed that there was a higher likelihood of triptan non-usage with a higher genetic effector score. Additionally, according to the logistic regression which took sex and age into account, females and older individuals are less likely to take triptans.

Though effector scores need to be validated in an independent cohort, we anticipate that these risk alleles will prove valuable for future studies. When combined with the positive association reported from previous literature, these data support the claim that there is a cumulative genetic contribution to triptan usage. In the future, with the accumulation of more effector alleles, a predictive genetic analysis could be made, with the aim of identifying patients with a low likelihood to respond to triptans.

When looking at clinical data, triptan users were more likely to have typical CH features such as having autonomic symptoms with their attacks, having alcohol as a headache trigger, and be current smokers (Table 3). Triptans are known to constrict blood vessels while smoking and alcohol have been known to have both vasodilatory and vasoconstrictive effects (Zhu and Parmley 1995; Kawano 2010; Nicolas and Nicolas 2022). It is worth noting that it is unclear if the vascular effects of triptans are the reason for the pharmacological effect or if it is a secondary effect of the main mechanism of action (May et al. 2001). Triptan users additionally tended to have a higher attack frequency than non-users contrary to the cohort of Giani et al. which found non-responders to have a higher attack frequency than responders (Giani et al. 2021). In our cohort it cannot be completely ruled out that high attack frequency could partly be due to medication-overuse headache in some cases. Giani et al. additionally found non-responders had a longer attack duration, when not applying treatment, than responders (100 min vs. 60 min in responders) (Giani et al. 2021). Although we did not find a significant difference in attack duration, this could be attributed to variances in data collection, as the Italian study employed more narrow time indications. In the study by Petersen et al., non-responders were more likely to be diagnosed with the chronic form of CH than responders, a result we could not replicate (Triptan users: 12.3%, Triptan Non-users: 12.1%, *P* = 1), nor Giani et al. (Giani et al. 2021). Petersen et al. conducted the study in a tertiary headache clinic and therefore had an overrepresentation of chronic individuals and refractory cases which could potentially explain the different results (Petersen et al. 2023).

A strength of this study is that only CH patients whose diagnosis is validated by a neurologist are included and both genetic and clinical characteristics are investigated. One of the limitations is the reliance on triptan usage as opposed to triptan response. Contraindications and side-effects can also lead to triptan non-usage which implies that our study groups might not be perfectly defined. Additionally, though different subtypes of triptans have similar mechanism of action, there are still CH patients who respond differently to different types of triptans, and we cannot account for the fact that study participants have not tried a triptan to which they are potentially responsive to. However, taking that into account, we still argue that there is a strong likelihood that regular usage and response are linked. The percentage of participants in this study who regularly took triptans is similar to the percentage of consistent responders previously reported (Law et al. 2013). In our study, classification of triptan usage vs non-usage is based on reported regular usage. Patients who specifically reported that they had tried triptans but were unresponsive to them were categorized as non-users.

The higher average age for triptans non-users may partially be due to contraindications such as hypertensions which is more common in the older population especially since when removing all individuals over the age of 60 from the analysis, the association between age and triptan usage is no longer significant. Though interestingly age of onset was also significantly younger in triptan users than those who did not use triptans. Additionally, age does not affect genotypic distribution and should not skew the genetic results. Potentially, people who carry these effector alleles could be less likely to be taking triptans if the same alleles correlate with a contraindication for triptans such as hypertension. However, we could not find any evidence that those SNPs are connected to hypertension in the published literature. A large GWAS conducted for hypertension did not detect rs1024905 as a risk SNP (Wang and Wang 2019). Schürks et al. found age to be a predictive factor for triptan response with triptan non-responders being older than responders in their German CH cohort additionally indicating non-response can be associated with an increased age (Schürks et al. 2007b). In future studies, it would be beneficial to investigate to what extent individuals do not take triptans due to side effects or contraindications.

## Conclusion

Our study shows that genetic variants such as rs1024905 can influence triptan usage in CH in Sweden. The cumulative effector score of five variants also indicates a complex genetic contribution to triptan usage. Additionally, significant associations with clinical features such as attack frequency and presence of autonomic symptoms indicate differences in disease profile between triptan users and non-users. Our data suggest that patients with a classic phenotype profile (being male, current smoker, having autonomic symptoms, and a relatively early onset) are more likely to regularly use triptans.

### Supplementary Information

Below is the link to the electronic supplementary material.

Supplementary file1 (PDF 44 KB)

## Data Availability

The data presented in this study are available on request from the corresponding author. The data are not publicly available due to privacy restrictions.
