# Supplementary material for: Genetic and Phenotypic Profiling of Triptan Users in a Swedish Cluster Headache Cohort
Source: J Mol Neurosci. 2024 Apr 18;74(2):45. doi: 10.1007/s12031-024-02219-1 (PMC11026232; doi:10.1007/s12031-024-02219-1)
Supplement: Supplementary file 1 — Supplementary file1 (PDF 44 KB) [file 12031_2024_2219_MOESM1_ESM.pdf]

Supplementary Data

Journal of Molecular Neuroscience

Genetic and Phenotypic Profiling of Triptan Users in a Swedish Cluster Headache Cohort

Felicia Jennysdotter Olofsgård [MSc. 1], Caroline Ran [PhD. 1], Yuyan Qin [BSc. 1], Carmen Fourier [PhD. 1], Elisabet Waldenlind [MD, PhD. 2,3], Anna Steinberg [MD, PhD. 2,3], Christina Sjöstrand [MD PhD. 2,4], Andrea Carmine Belin [PhD. 1*]

1. Centre for Cluster Headache, Department of Neuroscience, Karolinska Institutet, Stockholm, Sweden.
2. Department of Clinical Neuroscience, Karolinska Institutet, Stockholm, Sweden.
3. Department of Neurology, Karolinska University Hospital, Stockholm, Sweden.
4. Department of Neurology, Danderyd Hospital, Stockholm, Sweden.

*Corresponding author: andrea.carmine.belin@ki.se

Table S1: Detailed list of screened genetic variants

Genetic Variant	Gene	Minor Allele	Genotyping	Assay	Effector Allele Published	Effector Allele Sweden *
rs2651899	*PRDM16*	C	qPCR	C__27103073_10	T	T
rs6724624	*TRPM8*	G	qPCR	C__29368036_10	G	C
rs1024905	*FGF6/C12orf4*	G	qPCR	C___2768694_10	C	C
rs5443	*GNB3*	T	qPCR	C___2184734_10	C	C
5-HTTLPR	*5-HTTLPR*	Short	PCR-RFLP	NA	Short	Short

*PRDM16 = PR/SET Domain 16, TRPM8 = Transient Receptor Potential Cation Channel Subfamily M Member 8, FGF6 = Fibroblast Growth Factor 6, C12orf4 = Chromosome 12 Open Reading Frame 4 GNB3 = G Protein Subunit Beta 3, 5-HTTLPR = serotonin-transporter-linked promoter region, NA: not applicable, Minor Allele = minor allele in the general European population (H1000 Genomes Project Phase 3, gnomAD exomes database, and for 5-HTTLPR previous literature)[30]. Effector Allele Published = The more common allele in triptan non-responders reported in previous publications[6,7,14,16,17], Effector Allele Sweden = The more common allele in triptan non-users in Sweden, *Effector alleles used in the multi effector allele analysis were identified as the more common allele in triptan non-users in our cohort.*
